# Ongoing independent evolution of linezolid and vancomycin-resistance pELF-type linear plasmids across the One Health spectrum

**DOI:** 10.1128/aac.01168-25

**Published:** 2025-11-18

**Authors:** Yusuke Hashimoto, Duc Trung Dao, Ikuro Kasuga, Taichiro Takemura, Haruka Abe, Futoshi Hasebe, Takahiro Nomura, Jun Kurushima, Hidetada Hirakawa, Koichi Tanimoto, Pham Duy Thai, Hoang Huy Tran, Keigo Shibayama, Masato Suzuki, Haruyoshi Tomita

**Affiliations:** 1 Department of Bacteriology, Gunma University Graduate School of Medicine 12925, Maebashi, Gunma, Japan; 2 Department of Bacteriology, Nagoya University Graduate School of Medicine 12965, Nagoya, Aichi, Japan; 3 Research Center for Advanced Science and Technology, The University of Tokyo, Meguro-ku, Tokyo, Japan; 4 Department of Urban Engineering, Graduate School of Engineering, The University of Tokyo, Bunkyo-ku, Tokyo, Japan; 5 Institute of Tropical Medicine, Nagasaki University, Nagasaki, Nagasaki, Japan; 6 Laboratory of Bacterial Drug Resistance, Gunma University Graduate School of Medicine, Maebashi, Gunma, Japan; 7 Department of Medical Technology and Clinical Engineering, Gunma University of Health and Welfare 88270, Maebashi, Gunma, Japan; 8 National Institute of Hygiene and Epidemiology 310750, Hanoi, Vietnam; 9 Antimicrobial Resistance Research Center, National Institute of Infectious Diseases, Japan Institute for Health Security, Higashimurayama, Tokyo, Japan; Columbia University Irving Medical Center, New York, New York, USA

**Keywords:** *Enterococcus faecium*, vancomycin resistance, linezolid resistance, pELF-type linear plasmids, Tn*6674*-like transposon

## Abstract

Vancomycin and linezolid are crucial for treating enterococcal infections. The global emergence of linezolid- and vancomycin-resistant enterococci is a growing concern. Enterococcal pELF-type linear plasmids, which confer both types of resistance, have been identified worldwide from clinical and environmental sources. Phylogenetic and structural analyses of pELF-type linear plasmids revealed that they diverged into two distinct lineages and evolved through the integration of circular plasmids. Our findings on this ongoing evolution advance the fundamental knowledge required for the future development of global and cross-sectoral surveillance.

## INTRODUCTION

Vancomycin-resistant *Enterococcus faecium* (VREfm) is classified by the World Health Organization as a major threat with limited treatment options (1). Linezolid, a synthetic oxazolidinone, is a last-resort treatment for VREfm (2). Nevertheless, multiple mechanisms of linezolid resistance have been identified, including point mutations in the 23S rRNA domain V, the ABC-F family ribosomal protection protein genes *optrA* and *poxtA* (3–7), and the 23S rRNA methyltransferase gene *cfr* (8–10). The pELF-type linear plasmid has been increasingly reported worldwide in the clinical isolates of VREfm since its initial identification in 2019 and has been shown to contribute to antimicrobial resistance (AMR), including vancomycin resistance (11, 12). A pELF-type linear plasmid, carrying both the *optrA* gene and the *vanA* gene cluster, was recently reported through sentinel surveillance in the United States in 2024 (13). Similar pELF-type linear plasmids conferring dual resistance via *optrA* and *vanA* were also reported in India and Italy in 2022 and 2025, respectively (14, 15). Linezolid- and vancomycin-resistant enterococci (LVRE) harboring such plasmids are particularly concerning, as they exhibit multidrug resistance (MDR) to multiple last-resort antimicrobials, posing a serious global public health threat.

AMR is recognized as a global issue requiring a One Health approach, as AMR bacteria circulate between clinical, environmental, and animal sectors (16, 17). Low- and middle-income countries (LMICs) face heightened AMR risks due to limited AMR regulations and often inadequate sewage infrastructure (18). In this context, we identified a pELF-type linear plasmid co-carrying linezolid- and vancomycin-resistance genes in LVRE isolated from polluted urban sewage in Hanoi, Vietnam, a representative LMIC with severe AMR concerns. Of the 33 VREfm strains isolated in 2021, NUITM-VRE1 was identified as an LVREfm strain. Whole-genome sequencing (WGS) analysis revealed that this strain belonged to sequence type 117 (ST117) of clade A1 (Fig. S1). The complete genome consists of a 2.8-Mbp chromosome, a pELF-type linear plasmid designated as pELF_mdr, and six circular plasmids (accession nos. AP026772-AP026779) (Table S1). pELF_mdr, which is 139.0 kb in length, contained 182 coding sequences, including multiple AMR genes (ARGs), such as the *vanA* gene cluster, *poxtA2*, and *cfr(D),* along with the macrolide resistance gene *erm(B*), the fosfomycin resistance gene *fosB3*, and the florfenicol-chloramphenicol resistance gene *fexA* (Table S1). pELF_mdr could transfer to various *Enterococcus* spp., conferring linezolid, vancomycin, erythromycin, fosfomycin, and florfenicol-chloramphenicol resistance, transforming recipients into LVRE strains (Table S2).

**Fig 1:**
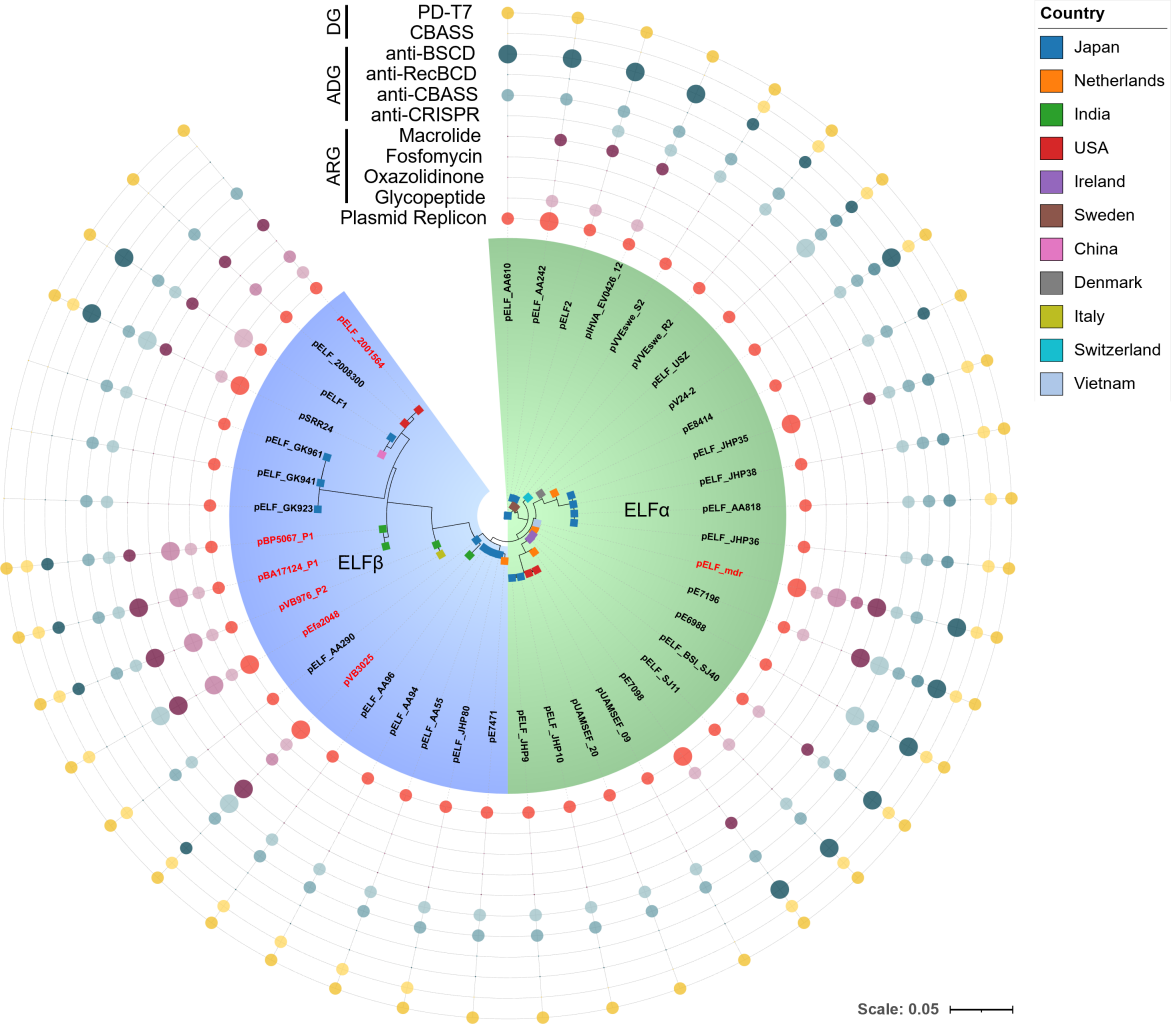
Phylogenetic meta-analysis of pELF-type linear plasmids. Core genes of the indicated 41 pELF-type linear plasmids in this study and in the public database were identified using Roary v3.13.0 (https://github.com/sanger-pathogens/Roary), and phylogenetic analysis was performed using RAxML v8.2.12 (https://github.com/stamatak/standard-RAxML) with 1,000 bootstrap replicates. The resulting phylogenetic tree, along with detection results of various ARGs, anti-defense genes (ADGs), and defense genes (DGs), was visualized using iTOL v7 (https://itol.embl.de). Dot size reflects copy number: small for a single hit, large for multiple hits; absence is not shown. Isolation countries are indicated by colored square symbols on each tree tip. Plasmid names in red indicate co-occurrence of genes conferring resistance to both oxazolidinone (including linezolid) and glycopeptide (including vancomycin). Green and blue backgrounds indicate ELFα and ELFβ lineages, respectively.

To investigate the evolutionary relationships and potential origins of these pELF-type linear plasmids capable of conferring linezolid and vancomycin resistance, a phylogenetic analysis was conducted based on core plasmid genes (Fig. 1; Table S3). All 41 analyzed pELF-type linear plasmids, including pELF_mdr identified in this study and those available in the public database, share 46 core genes, including the putative replication initiator protein gene *rep*_pELF_, the putative transfer-associated gene *ftsK*_pELF_, and the putative chaperone gene *clpX*_pELF_ (Table S4). The phylogenetic analysis revealed two distinct clusters, which we designated as ELFα and ELFβ (Fig. 1). pELF_mdr was found to belong to ELFα, whereas the *vanA* and *optrA*-carrying pELF-type linear plasmids, including those identified in the USA, India, and Italy, belonged to ELFβ (13–15), although they formed independent sub-clusters (Fig. 1).

pELF-type linear plasmids have been predominantly identified in *E. faecium*, and they encode a variety of defense genes (DGs) and anti-defense genes (ADGs), including toxin-antitoxin systems, broad-spectrum counter-defense systems, and anti-CRISPR systems (Fig. 1; Table S3) (12, 19). No specific ARGs or ADGs were perfectly conserved as core genes in 41 pELF-type linear plasmids; however, certain ADGs, particularly anti-CRISPR *acr* systems (87.8% of the total plasmids) and anti-CBASS systems (97.6%), were highly conserved, suggesting their potential roles in expanding the host range of pELF-type linear plasmid-mediated horizontal gene transfer.

In this study, two primary evolutionary pathways for pELF-type linear plasmids leading to linezolid resistance were identified: modification by transposons (Tn), such as Tn*6674*-like elements, and the integration of circular plasmids. The first pathway involves transposon-mediated acquisition of resistance genes. Except for pELF_mdr, the linezolid resistance gene carried on pELF-type linear plasmids was *optrA* (Fig. 1; Table S3). The *optrA* gene, predominantly found in *E. faecalis*, has spread globally since 2012 via Tn*6674* (20, 21). The *optrA* genes found on pELF_2001564, pELF_2008300, pVB3025, pBA17124_P1, and pBP5067_P1 were located on Tn*6674*-like elements, which shared structural similarities with the prototype Tn*6674* (Fig. S2A and B).

A BLAST search of the 10.5 kb Tn*6674*-like element containing *optrA* from pELF_2001564 revealed that 19 of 24 hit sequences with >80% query coverage were derived from *E. faecium* plasmids and chromosomes (Table S5). This suggests that the Tn*6674*-like element facilitates *optrA* spread specifically in *E. faecium*. In some cases, such as pVB976_P2 and pEfa2048, plasmids carried *erm* and *orf* but lacked the *tnpA/B/C* and *spc* genes characteristic of Tn*6674* (Fig. S2C). These *optrA*-carrying pELF-type linear plasmids have been identified in *E. faecium* isolates detected since 2017, suggesting that, similar to Tn*6674* in *E. faecalis*, they might have been acquired relatively recently. Phylogenetic analysis further indicates that these pELF-type linear plasmids may have independently acquired the *optrA*-containing Tn*6674*-like element (Fig. 1).

**Fig 2:**
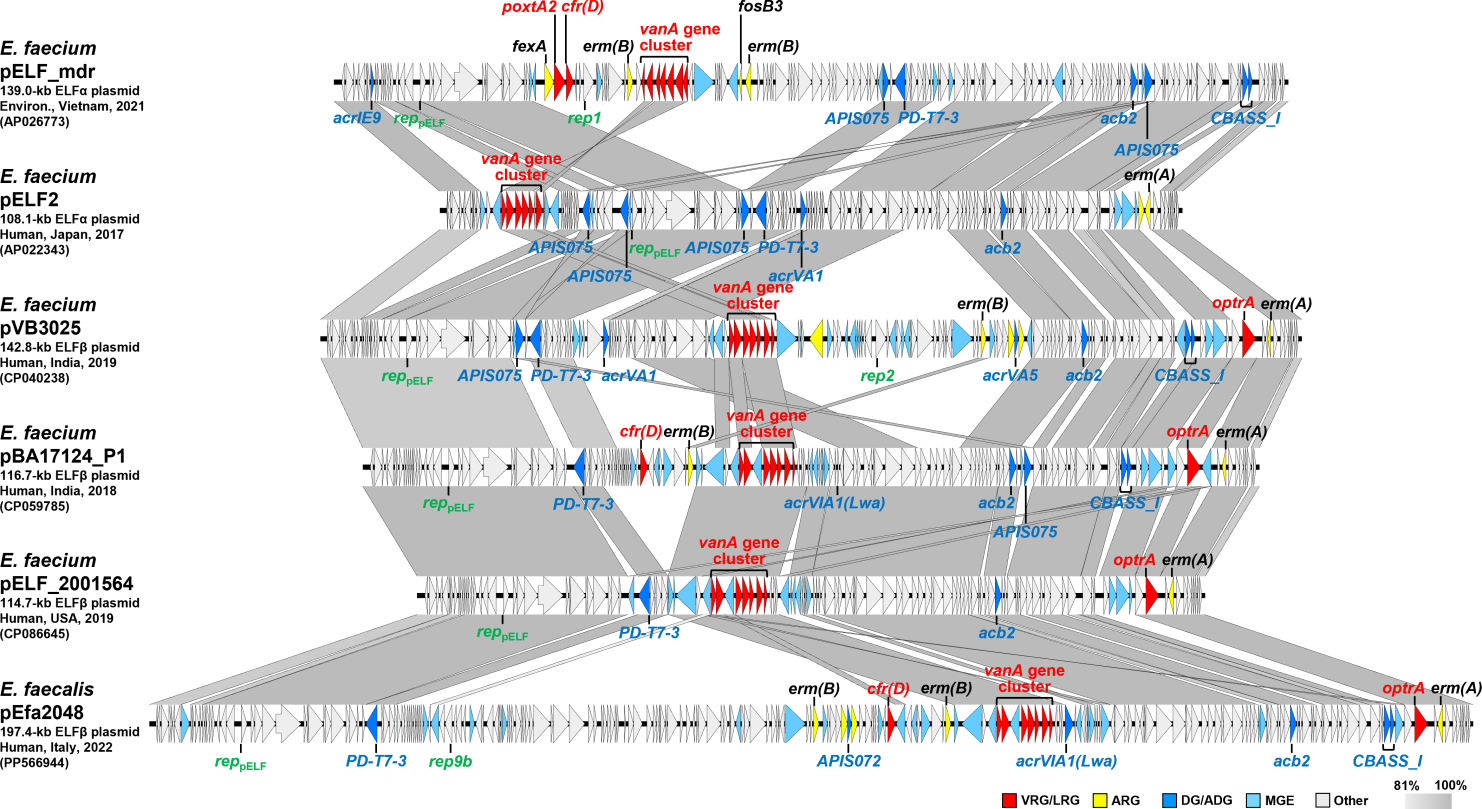
Comparison of genetic structures of pELF-type linear plasmids. Genetic comparative analysis was performed between the indicated six representative pELF-type linear plasmids from both ELFα and ELFβ lineages, carrying linezolid and vancomycin resistance genes, using EasyFig v2.2.2 (https://mjsull.github.io/Easyfig/), with synteny blocks colored according to the indicated sequence identity. Red indicates linezolid resistance genes (LRGs) and vancomycin resistance genes (VRGs), yellow indicates other ARGs, blue indicates anti-defense genes (ADGs) and defense genes (DGs), and light blue indicates mobile genetic elements (MGEs). ARGs, including VRGs and LRGs, ADGs, DGs, and plasmid replicons, were detected using ResFinder v4.7.2 (http://genepi.food.dtu.dk/resfinder), DefenseFinder v1.3.0 (https://github.com/mdmparis/defense-finder), dbAPIS v11/19/2024 (https://bcb.unl.edu/dbAPIS/), and PlasmidFinder v2.1 (https://cge.food.dtu.dk/services/PlasmidFinder/) with default settings, respectively.

The second pathway involves the integration of a circular plasmid, as seen in pELF_mdr, which gave rise to a linezolid and vancomycin-resistance plasmid. This plasmid carries *poxtA2* as its linezolid resistance gene and belongs to an independent plasmid cluster (Fig. 1 and 2). Analysis of the genetic structure of pELF_mdr revealed a 51.8 kb variable region (VR), which sequentially contains *fexA*, *poxtA2*, *cfr(D*), *erm(B*), the *vanA* gene cluster, *fosB3*, and *erm(B)* (Fig. 2; Fig. S3A). A BLAST search for this VR retrieved several enterococcal circular plasmids, including the 13.7 kb plasmid pIB-BOL isolated from *Enterococcus gallinarum* and the 33.5 kb plasmid pV386 isolated from *E. faecalis* (Fig. S3A) (6, 22). Similar to pELF_mdr, both pIB-BOL and pV386 carried *fexA* and *poxtA2*. In addition, pV386 carried *cfr(D*). Indeed, the VR of pELF_mdr contained replicon and transfer-related genes similar to those found in circular plasmids such as pV386, which do not appear to contribute to the function of the linear plasmid (Fig. S3A), suggesting that the VR originated from a circular plasmid.

Analysis of multi-replicon pELF-type linear plasmids confirms that the integration of circular plasmids is not a rare event in the evolution of linear plasmids (Fig. S3B and Table S6). Examination of the cointegrate junctions revealed that the integrated regions are flanked by directly oriented IS*1216E* elements, suggesting that these insertion sequences (IS) mediate the integration event. pELF-type linear plasmids frequently carry multiple copies of IS*1216E*, which provides a pre-existing foundation for high-frequency cointegrate formation through a targeted conservative mechanism (12, 23). The integrated circular plasmids were diverse, originating not only from *E. faecium* but also from *E. faecalis* and varied widely in size (Fig. S3). Furthermore, their origins were not limited to human sources, suggesting that pELF-type linear plasmids themselves may circulate through various sectors. This highlights the critical role of IS*1216E* in enabling these linear plasmids to acquire and accumulate a wide array of genetic modules from diverse ecological niches. Beyond ARGs, circular plasmid integration also serves as a vehicle for various fitness determinants, including a class II bacteriocin (lactococcin_972 family bacteriocin) gene and facilitates niche colonization (Fig. S3B). Furthermore, these integrated elements included both conjugative plasmids and non-conjugative plasmids (Fig. S3). This suggests that the integration of a circular plasmid effectively converts it into a module of the pELF-type linear plasmid, through which it gains horizontal transferability from the conjugative pELF backbone.

Our research demonstrates that pELF-type linear plasmids act as vehicles that confer the LVRE phenotype through a process defined by their remarkable flexibility in cointegrate formation. Through this mechanism, they can gather extensive sets of genes, including ARGs, in a single integration event. This capability is a primary driver behind the effective dissemination of linezolid and vancomycin resistance in *E. faecium* (23). Consequently, because such evolutionary events can emerge independently among different pELF-type linear plasmids, our findings advance the fundamental knowledge supporting the integration of global AMR surveillance efforts across the human, animal, and environmental sectors to identify these high-risk threats and enable effective intervention.

## Data Availability

The authors deposited all WGS data of *E. faecium* NUITM-VRE1 under the GenBank/EMBL/DDBJ accession numbers, AP026772 to AP026779.

## SUPPLEMENTAL MATERIAL

Supplemental figures aac.01168-25-s0001.docx Figures S1 to S3.

Supplemental tables aac.01168-25-s0002.xlsx Tables S1 to S6.

ASM does not own the copyrights to Supplemental Material that may be linked to, or accessed through, an article. The authors have granted ASM a non-exclusive, world-wide license to publish the Supplemental Material files. Please contact the corresponding author directly for reuse.
