# Supplementary material for: Ongoing independent evolution of linezolid and vancomycin-resistance pELF-type linear plasmids across the One Health spectrum
Source: Antimicrob Agents Chemother. 2025 Nov 18;69(12):e01168-25. doi: 10.1128/aac.01168-25 (PMC12691693; doi:10.1128/aac.01168-25)
Supplement: Supplemental figures — Figures S1 to S3. [file aac.01168-25-s0001.docx]

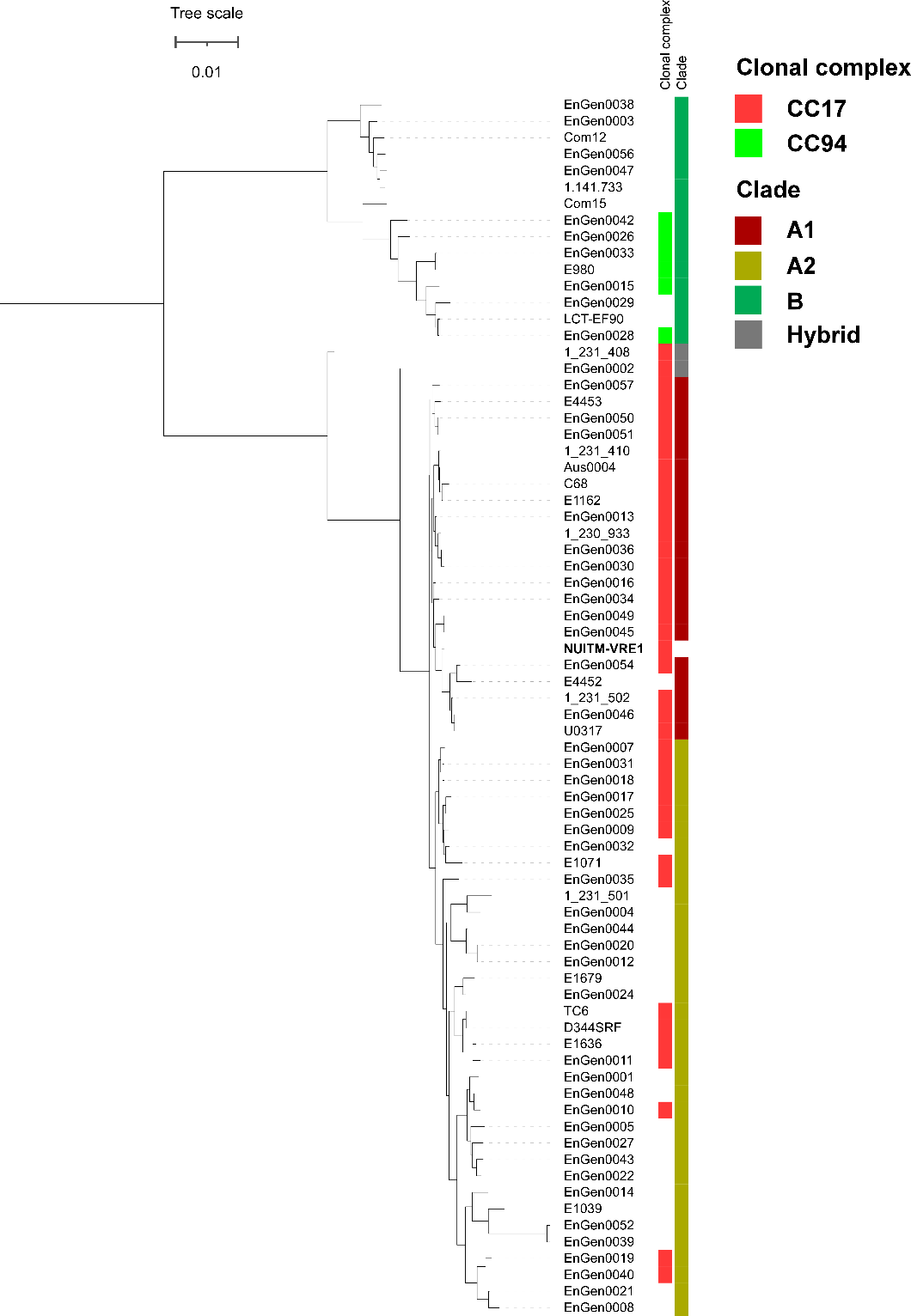


**Supplementary Figure S1. Phylogenetic analysis of *E. faecium* and *E. lactis* isolates.** Clade analysis of *E. faecium* NUITM-VRE1 in this study was performed using Roary v3.13.0 (https://github.com/sanger-pathogens/Roary), A phylogenetic tree of *E. faecium* (clade A1 and A2) and *E. lactis* (clade B) isolates in this study and in the public database was constructed using RAxML v8.2.12 (https://github.com/stamatak/standard-RAxML), and visualized using [iTOL](file:///C:\Users\Yusuke%20Hashimoto\Documents\細菌学\共同研究\感染研　鈴木先生\AMED_plasmid_ベトナム_JAC\ASM_241120-\Revise\20250919\iTOL) (1-3). Bold text indicates NUITM-VRE1. Clonal Complexes were assigned according to the pubMLST database, while clade classifications, including *E. faecium* (clade A1 and A2) and *E. lactis* (clade B), followed the previous report by Lebreton *et al* (4-7).


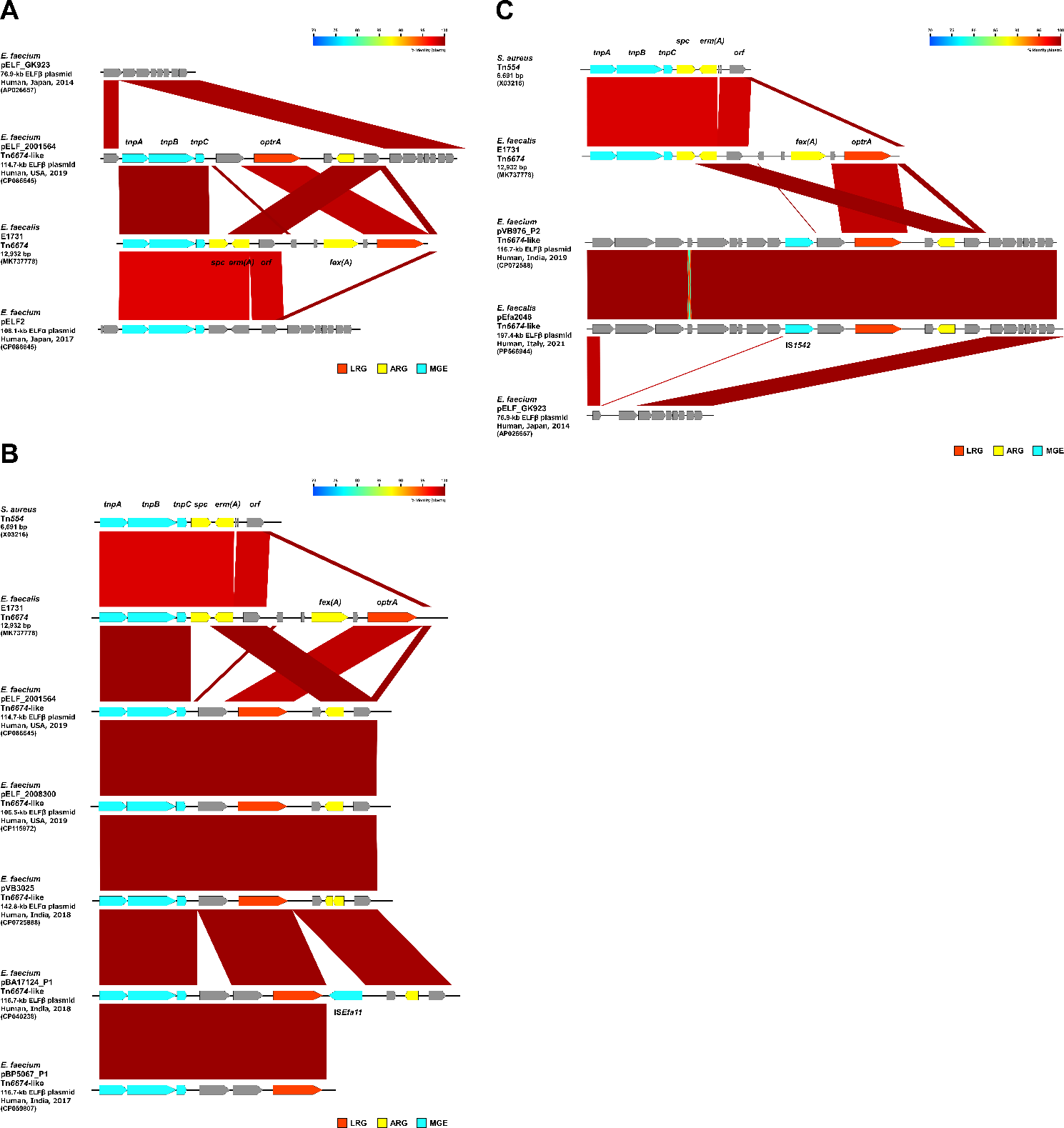


**Supplementary Figure S2**. **Comparison of ARG-associated elements on pELF-type linear plasmids.** A structural comparison of Tn*6674*-like elements associated with linezolid resistance genes (LRGs) on the indicated plasmids was performed and visualized using GenomeMatcher v3.09 (8). (A) The Tn*6674*-like element and its surrounding genetic structure in pELF_2001564 are schematically illustrated, with pELF_GK923 used as a reference. (B) The structures of Tn*6674*-like elements from pELF_2001564, pELF_2008300, pVB3025, pBA17124_P1, and pBP5067_P1 are compared. (C) The structures of Tn*6674* variants in pVB976_P2 and pEfa2048, which lack the *tnpA/B/C* and *spc* genes, are compared. Orange arrows indicate LRGs; yellow arrows indicate other ARGs; and light blue arrows indicate MGEs.


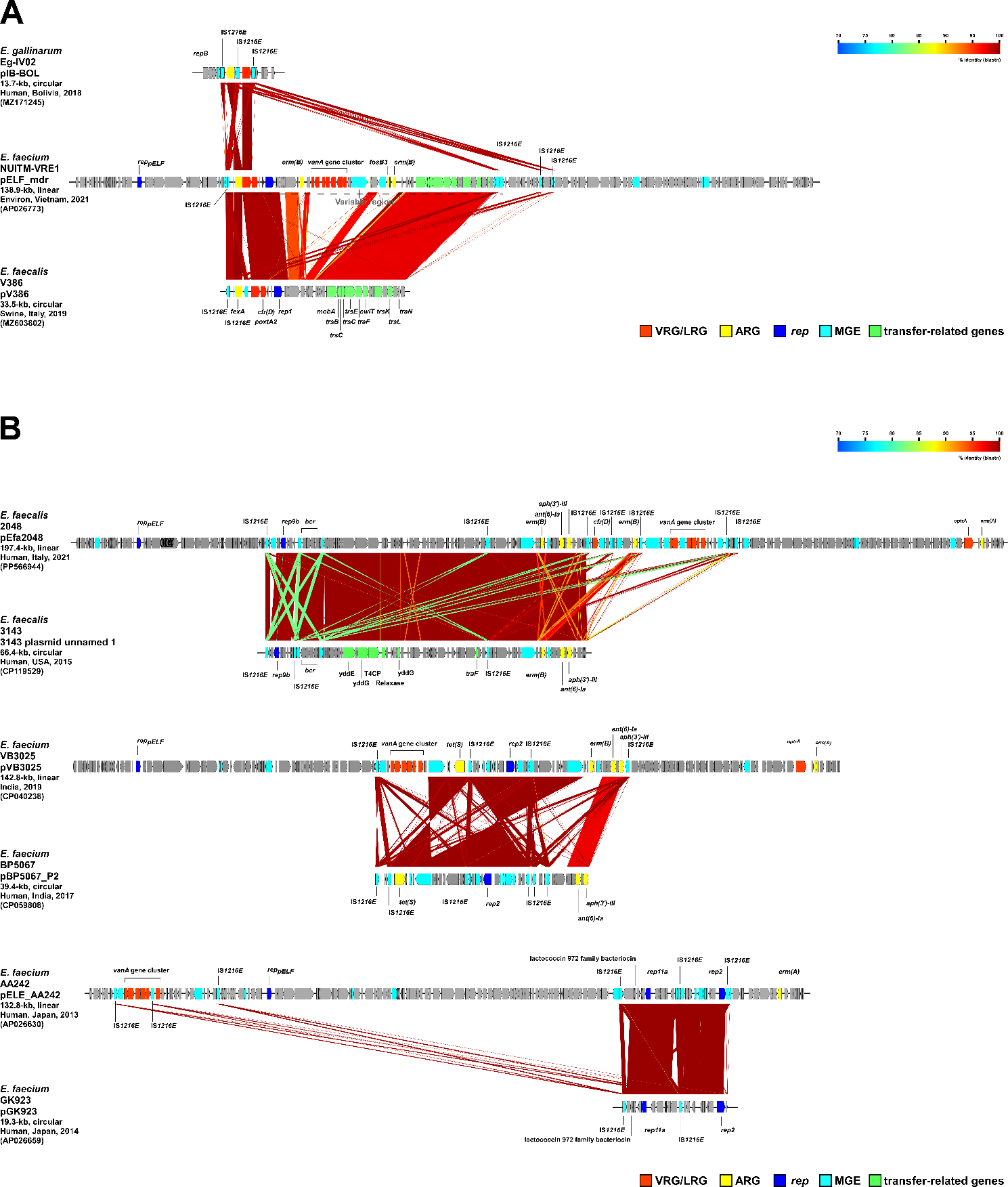


**Supplementary Figure S3**. **Comparison of pELF_mdr and circular plasmids in enterococci.** A structural comparison of pELF_mdr in this study and enterococcal circular plasmids in the public database was performed and visualized using GenomeMatcher v3.09 (8). (A) A structural comparison between the variable region of NUITM-VRE1 and the circular plasmids pV386 and pIB-BOL is shown. (B) A structural comparison between the multi-replicon pELF-type plasmids (pEfa2048, pVB3025, and pELF_AA242) and highly similar circular plasmids is shown.　Orange arrows indicate vancomycin resistance genes (VRGs) and linezolid resistance genes (LRGs); yellow arrows indicate other ARGs; light green arrows indicate transfer-related genes; and light blue arrows indicate MGEs. VR: variable region.

**Supplementary References**

1. Page AJ, Cummins CA, Hunt M, Wong VK, Reuter S, Holden MT, Fookes M, Falush D, Keane JA, Parkhill J. 2015. Roary: rapid large-scale prokaryote pan genome analysis. Bioinformatics 31:3691-3.

2. Stamatakis A. 2014. RAxML version 8: a tool for phylogenetic analysis and post-analysis of large phylogenies. Bioinformatics 30:1312-1313.

3. Letunic I, Bork P. 2024. Interactive Tree of Life (iTOL) v6: recent updates to the phylogenetic tree display and annotation tool. Nucleic Acids Research 52:W78-W82.

4. Jolley KA, Bray JE, Maiden MCJ. 2018. Open-access bacterial population genomics: BIGSdb software, the PubMLST.org website and their applications. Wellcome Open Res 3:124.

5. Lebreton F, van Schaik W, McGuire AM, Godfrey P, Griggs A, Mazumdar V, Corander J, Cheng L, Saif S, Young S, Zeng Q, Wortman J, Birren B, Willems RJ, Earl AM, Gilmore MS. 2013. Emergence of epidemic multidrug-resistant Enterococcus faecium from animal and commensal strains. mBio 4.

6. Kim E, Kim D-S, Yang S-M, Kim H-Y. 2022. The accurate identification and quantification of six Enterococcus species using quantitative polymerase chain reaction based novel DNA markers. LWT 166:113769.

7. Novais C, Almeida-Santos AC, Paula Pereira A, Rebelo A, Freitas AR, Peixe L. 2023. Alert for molecular data interpretation when using Enterococcus faecium reference strains reclassified as Enterococcus lactis. Gene 851:146951.

8. Ohtsubo Y, Ikeda-Ohtsubo W, Nagata Y, Tsuda M. 2008. GenomeMatcher: A graphical user interface for DNA sequence comparison. BMC Bioinformatics 9:376.
